# Minimally Invasive Spine Metastatic Tumor Resection and Stabilization: New Technology Yield Improved Outcome

**DOI:** 10.1155/2015/948373

**Published:** 2015-06-03

**Authors:** Ran Harel, Omer Doron, Nachshon Knoller

**Affiliations:** ^1^Spine Surgery Unit, Department of Neurosurgery, Sheba Medical Center, 52621 Ramat-Gan, Israel; ^2^Talpiot Medical Leadership Program, Sheba Medical Center, 52621 Ramat-Gan, Israel

## Abstract

Spinal metastases compressing the spinal cord are a medical emergency and should be operated on if possible; however, patients' medical condition is often poor and surgical complications are common. Minimizing surgical extant, operative time, and blood loss can potentially reduce postoperative complications. This is a retrospective study describing the patients operated on in our department utilizing a minimally invasive surgery (MIS) approach to decompress and instrument the spine from November 2013 to November 2014. Five patients were operated on for thoracic or lumbar metastases. In all cases a unilateral decompression with expandable tubular retractor was followed by instrumentation of one level above and below the index level and additional screw at the index level contralateral to the decompression side. Cannulated fenestrated screws were used (Longitude FNS) and cement was injected to increase pullout resistance. Mean operative time was 134 minutes and estimated blood loss was minimal in all cases. Improvement was noticeable in neurological status, function, and pain scores. No complications were observed. Technological improvements in spinal instruments facilitate shorter and safer surgeries in oncologic patient population and thus reduce the complication rate. These technologies improve patients' quality of life and enable the treatment of patients with comorbidities.

## 1. Introduction

Spine metastases involving the epidural compartment and resulting in spinal cord compression are often best treated operatively [1]. Multiple approaches for surgical treatment of spinal metastases have been described; however, no superiority of one technique over the other has been demonstrated [1–4]. Patients harboring spinal metastases are often compromised by multiple medical conditions such as anemia, immunodeficiency, tumor related osteoporosis, pain intolerance, and chronic infections [2]. These conditions subject patients operated on to increased risk as the standard open spine surgery involves significant blood loss, high wound infection rates especially if these levels were irradiated previously, risk of hardware failure, need for intense pain management, and infection related complications [5–7]. Instrumentation of the osteoporotic spine can be managed by long instrumentation constructs, but these increase operative time and hemorrhage and elongate the lever arm on the construct terminal end causing increased pullout forces on the terminal screws. Long constructs increase the stiffness of the operated region, thus increase motion in adjacent levels, and may cause failure in these levels [8]. Increase in screw pullout resistance in conjunction with shorter constructs can be achieved by injection of Polymethylmethacrylate (PMMA) through fenestrated screws [9, 10]. Technological advances allow spine surgeons to decompress the spinal cord and nerves through small incisions using tubular retractors and microscopic visualization and to stabilize the spine with percutaneous screw insertion. These techniques are used regularly by a growing number of surgeons for degenerative pathologies [11–13]. Minimally invasive surgery for metastases minimizes hemorrhage and wound complications and reduces hospital stay and narcotic consumption [14–16].

This paper describes the authors' experience utilizing minimally invasive retractors for decompression of the spinal cord and percutaneous cannulated fenestrated screws for short segment PMMA augmented instrumentation.

## 2. Materials and Methods

This is a retrospective study of patients records who were operated on in Sheba medical center neurosurgical spine unit. After the study was approved by the Sheba Institutional Review Board, the authors reviewed the records of patients operated on from November 2013 to November 2014. We evaluated patients' demographics and medical condition before and after surgery, imaging data, operative and postoperative management, complications, and functional status. Data was collected from patients' medical files and imaging studies.

### 2.1. Surgical Technique

**Figure 1:**
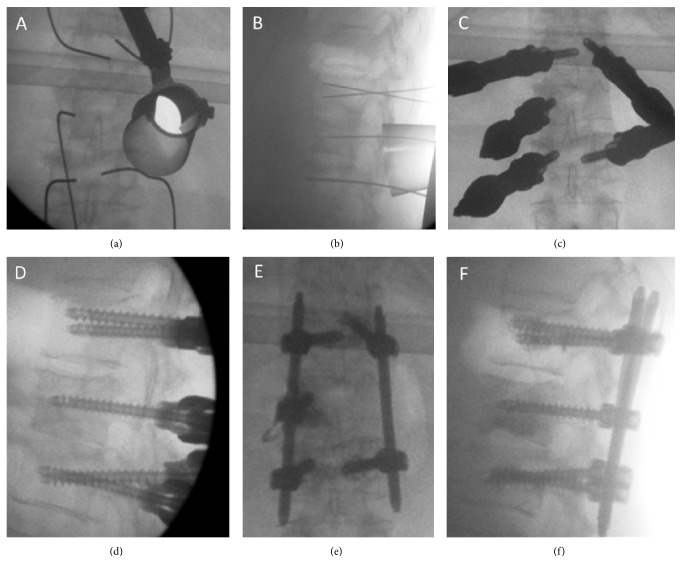


Patients were anesthetized and intubated, placed prone on a radiolucent table. Following preparation and draping, utilizing fluoroscopic guidance the surgeon (RH) inserted percutaneous K-wires to the level above and below the index vertebrae and to the index vertebrae contralateral to the decompression side. The decompression side was determined according to the CT and MRI scan in order to achieve maximal cord decompression and tumor resection. A minimally invasive expandable tubular retractor was introduced using a percutaneous approach over the facet, lamina, and transverse process on the decompression side and opened under fluoroscopic guidance (X-tube, Metrix, Medtronic, USA) (Figures 1(a) and 1(b)). Using a high speed drill and the transpedicular approach the thecal sac was exposed and decompressed and partial corpectomy was achieved. The retractor was retrieved and percutaneous insertion of cannulated fenestrated screws over the previously inserted K-wires followed (Longitude FNS system, Medtronic, USA) (Figures 1(c) and 1(d)). Under fluoroscopic imaging PMMA was injected through the screws (1.5 cc per screw). Percutaneous rod insertion and locking followed (Figures 1(e) and 1(f)). Wounds were irrigated and closed and patients were transferred to the recovery room.

## 3. Results

Over the recent year (November 2013 to November 2014) 5 patients had undergone minimally invasive decompression and percutaneous stabilization of the spine.  Table 1 summarizes patients' demographic details, pathological diagnosis, surgical and radiation treatment, and complications. Mean age was 57, 2 patients were ambulatory, 2 patients could walk with assistance, and 1 was wheel-chair bound. In 4 patients the indication for surgery was spinal canal compromise with compression of the cord or nerves and in 1 patient the indication was recurrence of solitary cholangiocarcinoma metastases following 3D radiotherapy with a total dose of 64 Gy. Two patients were treated for lower thoracic region and 3 patients were operated on in the upper lumbar region. Tumor origins were from the colon, nasopharynx, cholangiocarcinoma, and 2 bladder carcinomas. Four patients had 1-level hemicorpectomy using a minimally invasive expandable tubular retractor system (X-TUBE, METRX, Medtronic, USA) with a short construct instrumentation using Longitude FNS system (Medtronic, USA) augmented with a screw on the index level contralateral to the corpectomy approach and polymethyl methacrylate (PMMA) was injected to through all screws (Figures 1(e) and 1(f)). In one case, a 2-level decompression was accomplished utilizing a left sided approach to D9 vertebra and a right sided approach to D10, with instrumentation ranging from D8 to D11 with PMMA augmented screws. Intraoperative bleeding was minimal in all patients. There were no intraoperative complications. Mean operative time was 134 minutes (range: 110–177). All patients had a postoperative CT scan on postoperative day 1 demonstrating the following results: 2 patients with 5 out of 5 screws with no breach, 2 patients with 1/5 screws with 2 mm medial breach (grade 1 [17] asymptomatic was not revised) and 2 of the patients had 2/2 screws with 2 mm medial breach (grade 1 [17] asymptomatic was not revised). PMMA was seen in all cases around the screw tip, minimal cement leakage to the vertebral lateral border was noticed in one patient, and the rest had no cement leakage. Three patients were discharged home with a mean length of stay of 4 days (range 4-5 days). One patient was transferred to the oncology department for chemotherapy and the other was discharged to rehabilitation facility after 10 days. Two patients were treated with radiation therapy prior to surgery and were operated on when the radiation therapy failed. One patient was treated with fractionated radiation after the surgery and 2 were treated with spine radiosurgery following the surgery. None of the patients developed wound complications or hardware failure.

**Figure 2:**
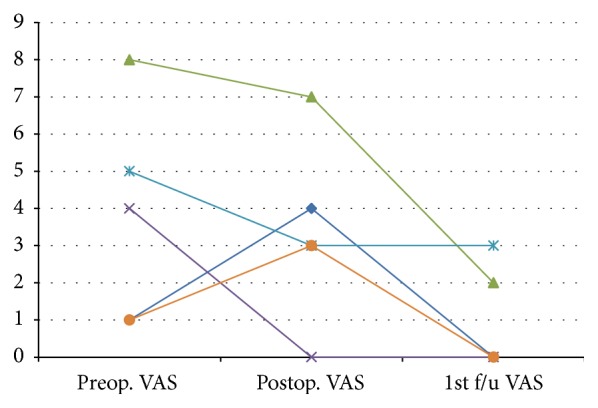
Visual analogue scale (VAS) is presented for each patient before the surgery, immediately after the surgery and during follow-up.

**Figure 3:**
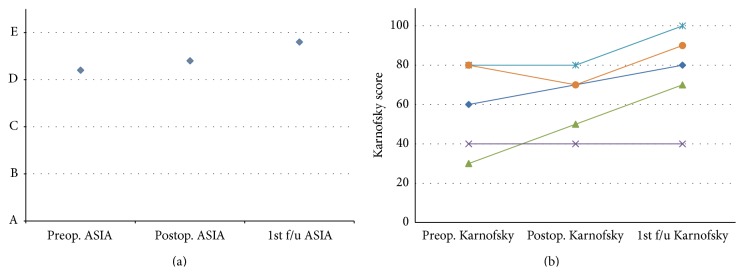
Mean ASIA score (a) and patients' Karnofsky score (b) as recorded before the surgery, immediately after the surgery, and during follow-up.

On admission 2 patients were ambulating, 2 were ambulating with assistance, and 1 was wheelchair bound. On discharge, 3 patients were ambulatory and 2 were ambulatory with assistance.  Figure 2 demonstrates the improvement in pain assessed with the visual analogue scale (VAS). Asia scale and Karnofsky performance scale are presented in Figures 3(a) and 3(b) accordingly. No mortality was observed during the first 3 months after surgery. No other late complications were observed.

## 4. Discussion

Surgical management of spinal metastases has been shown to be effective in selected cases that were operated on utilizing an approach according to surgeons' discretion [1]. Minimizing surgical extent while achieving surgical goals can improve outcomes and reduce complication in spinal metastases surgery. Multiple publications describe the attempt to minimize surgical collateral damage in anterior thoracic spine surgery, a long established approach to decompress anterior lesions [4, 14, 18, 19]. In recent years the posterolateral approach has gained popularity allowing surgeons to decompress the spinal cord and instrument the spine using the same incision while avoiding the transthoracic approach related complications. However, this approach is accomplished through a long posterior incision harboring significant risk for major blood loss and wound complications [4, 14]. Minimally invasive technology, including tubular retractors and percutaneous pedicle screws, have evolved in recent years mainly for the treatment of degenerative spine pathologies, enabling safer approaches to spine tumors [20, 21]. Ten metastatic patients described by Zairi et al. [15] had undergone spinal cord decompression utilizing expandable tubular retractor and percutaneous pedicular screws stabilization showing neurological improvement in 80% of the patients and only 1 urinary tract infection complication. In this series all patients were instrumented 2 levels above and below the treated level, mean estimated blood loss was 400 mL, and mean operative time was 170 minutes. In the series we describe that the decompression was performed through a unilateral approach using expandable tubular retractor limiting blood loss and surgical incision, and shorter reinforced constructs were used; hence, estimated blood loss was minimal and mean operative time was 36 minutes shorter. Longer constructs increase the stiffness of the spine and allow for stresses to be distributed between more screws [22]. However, longer constructs increase operative time and intraoperative bleeding, thus increasing infection risks [6]. Longer constructs are advocated in traumatic unstable vertebral fractures in order to increase the stability and stiffness across the fracture [22]. Vertebras adjacent to a traumatic fracture usually sustain normal architecture and pullout resistance, while many metastatic patients suffer from reduced pullout resistance due to multiple metastases, prior radiation, and osteoporosis. Long constructs have increased lever arm and stiffness; thus, they increase the stress on the terminal screws and may result in junctional kyphosis [23]. In the current series we used a short construct design in order to reduce the stress on the terminal screws. In order to increase the pullout resistance of all screws, we used fenestrated screws and injected bone cement into all operated levels. Biomechanical evaluation of screws augmented with cement demonstrated a 1.5–2.5-fold increase in pullout resistance [10, 24]. In all the described cases we instrumented the index level on the contralateral side to the decompression and injected bone cement to the vertebral body residual. This reinforced residual adds to the load bearing efficacy of the construct, thus reducing the chance of construct failure [25, 26]. Biomechanical evaluation of short constructs utilizing screws at the fractured level demonstrated increased stiffness and stability across the fracture [27, 28]. These evaluations used bilateral screws at the injured site. In the current study, a unilateral screw was inserted at the index level in order to gain more stability as the other side was resected. The addition of an intermediate screw at the index level converts the construct from a bridging implant (with only terminal screws) to a three-point bending construct. This provides significant biomechanical advantage, by the addition of three-point bending forces to the complex mechanical milieu [29].

Finally, a relatively short life expectancy may favor a short, rather than a long, construct. The benefits associated with long fixation usually accrued over the long term. Such may not be the case in many metastatic spine tumor patients. This is corroborated by the fact that during mean 5-month follow-up, all constructs remained stable.

## 5. Conclusions

Advances in minimally invasive decompression and instrumentation can facilitate better surgical results in metastatic spine patients, utilizing shorter constructs and thus minimizing operative time, operative bleeding, and surgical complications.

## Figures and Tables

**Table 1:** Patients' demographics and surgical treatment.

Patient number	Age	Sex	Primary tumor	Surgery	Surgical complications	Estimated blood loss	Sequence treatment
1	54	Female	Cholangiocarcinoma	Right D9 hemicorporectomy, left D10 hemicorporectomy D8–D11 percutaneous instrumentation	None	Minimal	Preoperative fractionated radiation

2	60	Male	Bladder carcinoma	Right L1 hemicorporectomy, D12–L2 percutaneous instrumentation	None	Minimal	Preoperative fractionated radiation

3	82	Female	Bladder carcinoma	Left L2 hemicorporectomy, L1–L3 percutaneous instrumentation	None	Minimal	Postoperative fractionated radiation

4	49	Female	Nasopharyngeal adenocarcinoma	Left D9 hemicorporectomy, D8–D10 percutaneous instrumentation	None	Minimal	Postoperative stereotactic radiation

5	41	Female	Colon carcinoma	Left L2 hemicorporectomy, L1–L3 percutaneous instrumentation	None	Minimal	Postoperative stereotactic radiation
